# Hybrid Ag nanowire transparent conductive electrodes with randomly oriented and grid-patterned Ag nanowire networks

**DOI:** 10.1038/s41598-017-11964-w

**Published:** 2017-09-14

**Authors:** Bonhee Ha, Sungjin Jo

**Affiliations:** School of Architectural, Civil, Environmental, and Energy Engineering, Kyungpook National University, Daegu, 41566 Korea

## Abstract

To improve the electrical properties of silver nanowire (Ag NW) transparent conductive electrodes (TCEs), the density of Ag NW networks should be increased, to increase the number of percolation paths. However, because of the inverse relationship between optical transmittance and electrical resistivity, the optical properties of Ag NW TCEs deteriorate with increasing density of the Ag NW network. In this study, a hybrid Ag NW electrode composed of randomly oriented and grid-patterned Ag NW networks is demonstrated. The hybrid Ag NW electrodes exhibit significantly improved sheet resistances and slightly decreased transmittances compared to randomly oriented Ag NW networks. Hybrid Ag NW TCEs show excellent mechanical flexibilities and durabilities in bending tests with a 5 mm radius of curvature. Moreover, flexible transparent film heaters (TFHs) based on the hybrid Ag NW electrodes show elevated maximum temperatures relative to TFHs based on randomly oriented Ag NW electrodes, when operated at the same input voltages.

## Introduction

Transparent conductive electrodes (TCEs) are essential components of optoelectronic devices such as touch panels, solar cells, organic light emitting diodes, and smart windows^1–4^. Recent advances in technology have increased the interest in flexible and stretchable devices, and hence in the research on TCEs with respect to flexibility and stretchability, in a wide variety of application in electronics. Because of their high transparencies and low sheet resistances, TCEs based on indium tin oxide (ITO) are currently the most widely used materials. However, application of ITO in emerging flexible and stretchable electronic devices is challenging because of its vulnerability to mechanical deformation^5, 6^. Many studies have focused on various TCE materials such as silver nanowires (Ag NWs), carbon nanotubes, graphenes, and conductive polymers to replace ITO^7–10^. Among these, Ag NWs have attracted the most attention because of their superior electrical conductivities and optical transmittances. Moreover, using Ag NWs is advantageous because they can be prepared by low-cost, solution-based processes such as spin-coating, drop-casting, rod-coating, and spray-coating. However, to apply Ag NW electrodes in high performance electronic devices, it is necessary to develop techniques that can reduce sheet resistance while maintaining high transmittance.

The electrical properties of a NW network are determined by its nature, such as the length and diameter of NWs, as well as the NW density^11, 12^. Electrical conduction in a NW network is affected by the resistances at junctions of NWs, because of the percolative nature of conduction. As the diameters of the NWs decrease, the resistance of the NW network increases because of the increased resistances of individual NWs. As the length of the NW increases, the conduction path could be achieved with a smaller number of NWs, and the number of junctions between the NWs could be reduced, thereby decreasing the resistance of the NW network. Methods such as thermal annealing, optical sintering, electron-beam exposure, and mechanical pressing have been proposed to reduce junction resistances between NWs, but drawbacks to these methods include the limited availability of substrates that can withstand high temperatures as well as the need for complex and expensive equipment^13–16^. In addition, the density of NWs plays an important role in determining the conduction and optical properties of the NW network. In general, increasing the density of NWs to improve the electrical conductivity of the NW network can lead to decreased transmittance of the NW network because of their inverse relationship. Therefore, it is imperative to develop newer technologies that can reduce the resistances of NW networks without affecting their transmittances.

In this study, to overcome the trade-off relationship between electrical conductivity and optical transmittance in a randomly oriented Ag NW network, we introduced a new approach to fabricating hybrid Ag NW networks, comprising randomly oriented and grid-patterned Ag NWs. Ag NWs deposited by a conventional coating process have a random network structure. Therefore, to improve their electrical conductivity, the density of Ag NWs should be increased to increase the number of contact junctions required for electrical percolation. However, increasing the densities of Ag NWs can adversely affect the transmittances of the derived TCEs. However, hybrid Ag NW electrodes with random and grid-patterned Ag NW networks developed in this study can provide an adequate number of electrical percolation paths with low density of the random Ag NW network, because the grid-patterned Ag NW network yields a regular electrical path^17^. To fabricate the hybrid Ag NW electrode, Ag NW line patterns of various line widths with 100 µm spacing were obtained by a micromolding-in-capillary (MIMIC) process. Based on an optimized MIMIC process, a technique for fabricating Ag NW grid patterns was newly developed using two consecutive MIMIC processes, in which the directions of the micro-channels were perpendicular to each other.

## Results and Discussion

**Figure 1:**
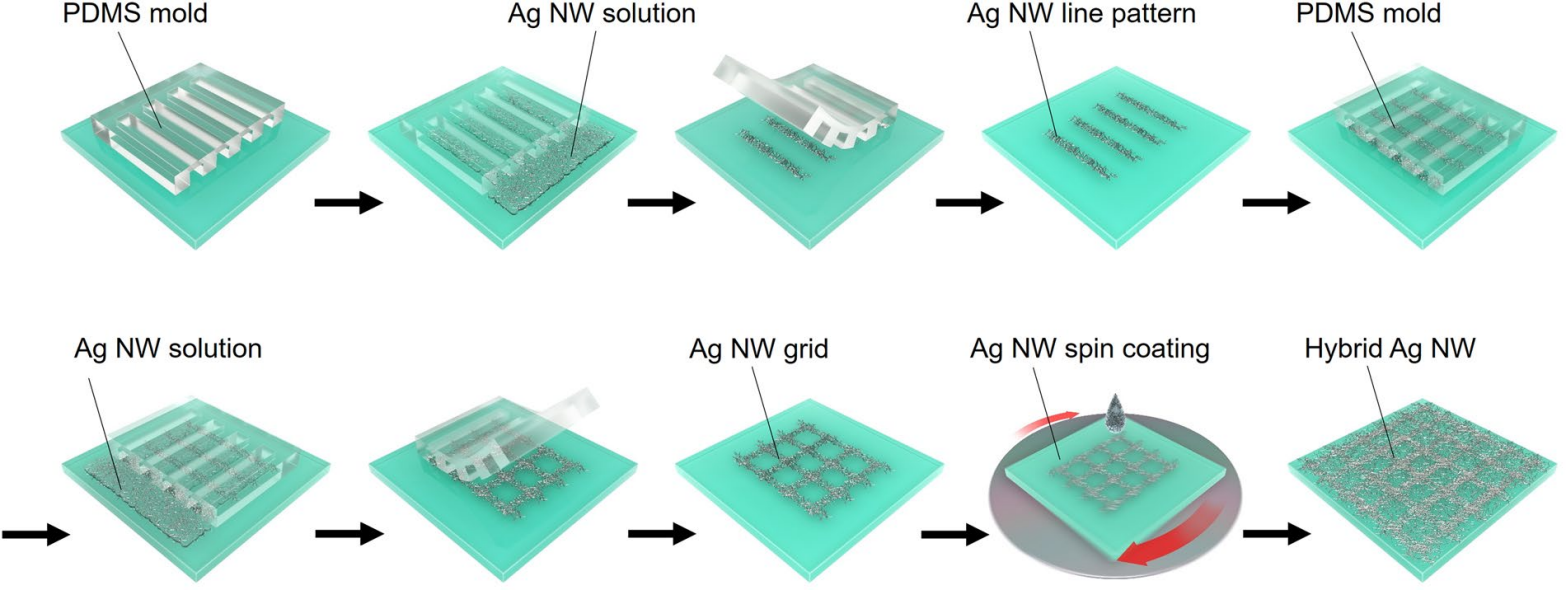
Schematic illustration of the steps involved in fabricating hybrid Ag NW electrodes.

Figure 1 shows a schematic illustration of the procedure used for the fabrication of hybrid Ag NW networks. An SU-8 layer used as a master mold was spin-coated on a Si wafer and patterned with the desired line and space intervals by photolithography. To fabricate a replicated polydimethylsiloxane (PDMS) mold with micro-channels, PDMS was cast on a master mold, as shown in Figure S1 in the supplementary information (SI). A MIMIC technique was adopted to obtain Ag NW grids. The PDMS mold was contacted face-down on the patterned substrate surface to form micro-channels, and a drop of the Ag NW suspension was introduced at the PDMS mold tip. The Ag NW suspension filled the micro-channels of the PDMS mold through capillary forces. A vertically-aligned Ag NW line pattern was obtained by releasing the PDMS mold. To fabricate the Ag NW grid pattern, the micro-channel of the PDMS mold was placed on the substrate in a direction perpendicular to the previously-formed Ag NW line pattern, and the MIMIC process was repeated to form a horizontally-aligned Ag NW line pattern overlaid on the first one. Finally, a hybrid Ag NW electrode comprising random and grid networks was fabricated by spin-coating an Ag NW suspension onto the Ag NW grid.

**Figure 2:**
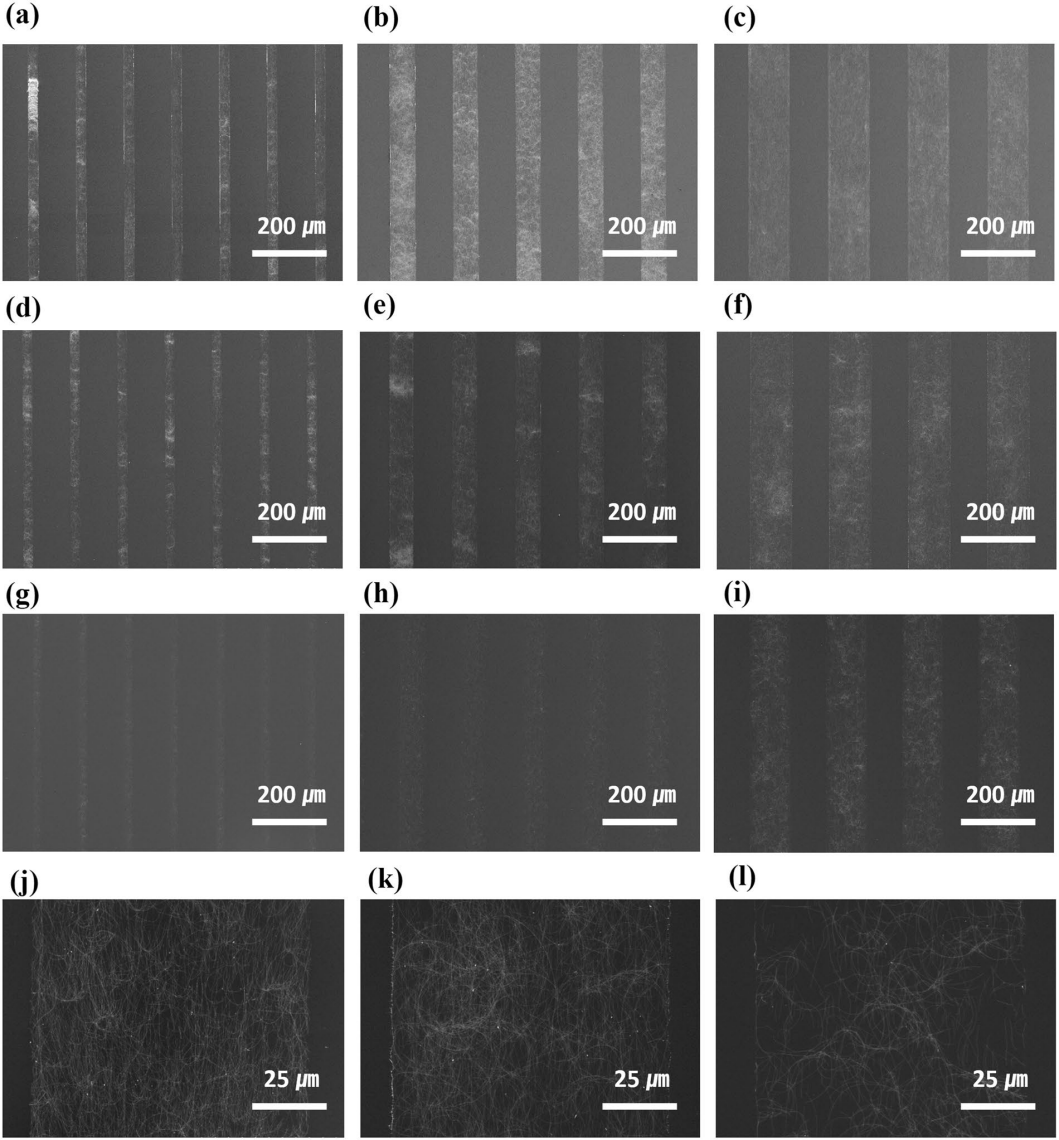
SEM images of Ag NW line patterns with 20, 60, and 100 µm line widths fabricated with (**a**–**c**) 0.2 wt% Ag NW suspension, (**d**–**f**) 0.1 wt% Ag NW suspension, and (**g**–**i**) 0.05 wt% Ag NW suspension. (**j**–**l**) Magnified SEM images corresponding to (**c**,**f**, and **i**).

In the MIMIC process, the rate of capillary filling depends on the kinematic viscosity, surface tension of the liquid, radius of the capillary, and length of the filled section of the capillary^18, 19^. To investigate the effect of the radius of the capillary on Ag NW pattern formation, we investigated the morphologies of Ag NW line patterns formed by changing the heights and widths of micro-channels. To examine the effect of micro-channel height, PDMS molds with micro-channel heights of 7 and 25 µm were replicated from SU-8 master molds using SU-8 2007 and 2025, respectively (Figure S1, SI). Greater height of the micro-channel leads to faster filling of the Ag NW suspension in the micro-channel, because the radius of the capillary increases. However, as the height of the micro-channel increased, larger quantity of Ag NW suspension filled the micro-channels, resulting in both increased Ag NW density in the Ag NW patterns and the formation of aggregates at the micro-channel walls (Figure S2, SI). Therefore, a PDMS mold with micro-channel height of 7 µm was used for the MIMIC process in this study. Further, to investigate the effect of the width of the micro-channel on the morphology of the Ag NW line pattern, the MIMIC process was performed using a PDMS mold with micro-channel widths of 20, 60, and 100 µm. Figure 2 shows scanning electron microscopy (SEM) images of these Ag NW line patterns as functions of the width of the micro-channel and concentration of the Ag NW suspension. Although Ag NW patterns were uniformly formed at micro-channel widths of 60 and 100 µm, when the width was reduced to 20 µm, some aggregation of Ag NWs was observed in the Ag NW patterns, as shown in Fig. 2(a). To resolve this, the concentration of the Ag NW suspension was decreased from 0.2 wt% to 0.1 and 0.05 wt%. As shown in Fig. 2(g–i), uniform Ag NW patterns could be obtained at all micro-channel widths when 0.05 wt% Ag NW suspensions were used. Figure 2(j–l) shows that the Ag NW density decreased in the Ag NW line pattern formed with 100 µm wide micro-channels, as the concentration of the Ag NW suspension decreased.

**Figure 3:**
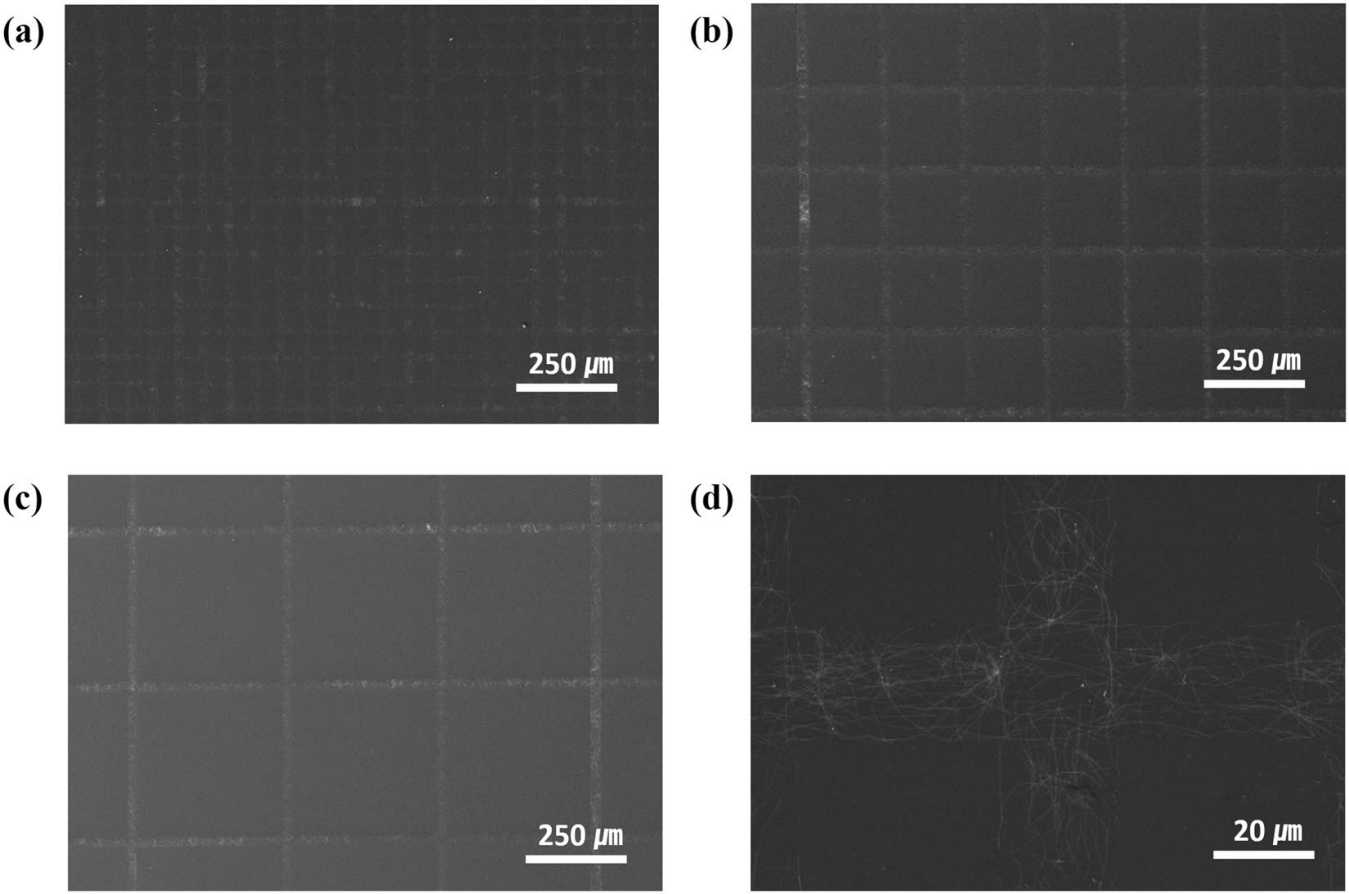
SEM images of Ag NW grid patterns with grid width of 20 µm and grid pitches of (**a**) 50 µm, (**b**) 200 µm, and (**c**) 400 µm. (**d**) Magnified SEM image of (**a**).

In general, the optoelectronic characteristics of micro-grid electrodes are dependent on the fill factor, which represents the ratio of the area of the grid pattern to the total area of the electrode^20^. Since the fill factor is determined by the width of the grid and the grid-to-grid distance (grid pitch), the Ag NW grid was fabricated by varying the grid pitch dimension to 50, 200, and 400 µm, with a minimum grid width of 20 µm (Fig. 3), to optimize the fill factor of the Ag NW grid. As shown in Fig. 3(a), a uniform Ag NW grid was fabricated using 0.05 wt% Ag NW suspension. However, when 0.3 wt% Ag NW suspension was used, aggregation of Ag NWs occurred at the grid intersection during the second MIMIC process, resulting in an uneven grid pattern (Figures S2(c), S2(d); SI).

To optimize the optoelectronic properties of the hybrid Ag NW electrodes, the randomly oriented and grid-patterned Ag NW networks should be investigated separately, since the optoelectronic characteristics of the hybrid electrodes are affected by both patterns of Ag NW networks. First, to investigate the optoelectronic characteristics of the hybrid Ag NW electrode with respect to the change in fill factor of the grid pattern, Ag NWs were spin-coated on Ag NW grids with grid pitch dimensions of 50, 200, and 400 µm to fabricate Ag NW electrodes, in order to obtain hybrid networks composed of both randomly oriented and grid-patterned networks. The transmittances and sheet resistances of spin-coated and hybrid Ag NW electrodes are summarized in Table S1, SI. Table S1, SI shows that the electrical property of the hybrid Ag NW electrode was enhanced at the expense of its optical property. As the grid pitch decreased from 400 µm to 50 µm, the transmittance of the hybrid electrode decreased slightly (0.27%), but the sheet resistance of the hybrid electrode decreased significantly (29.94%).

**Figure 4:**
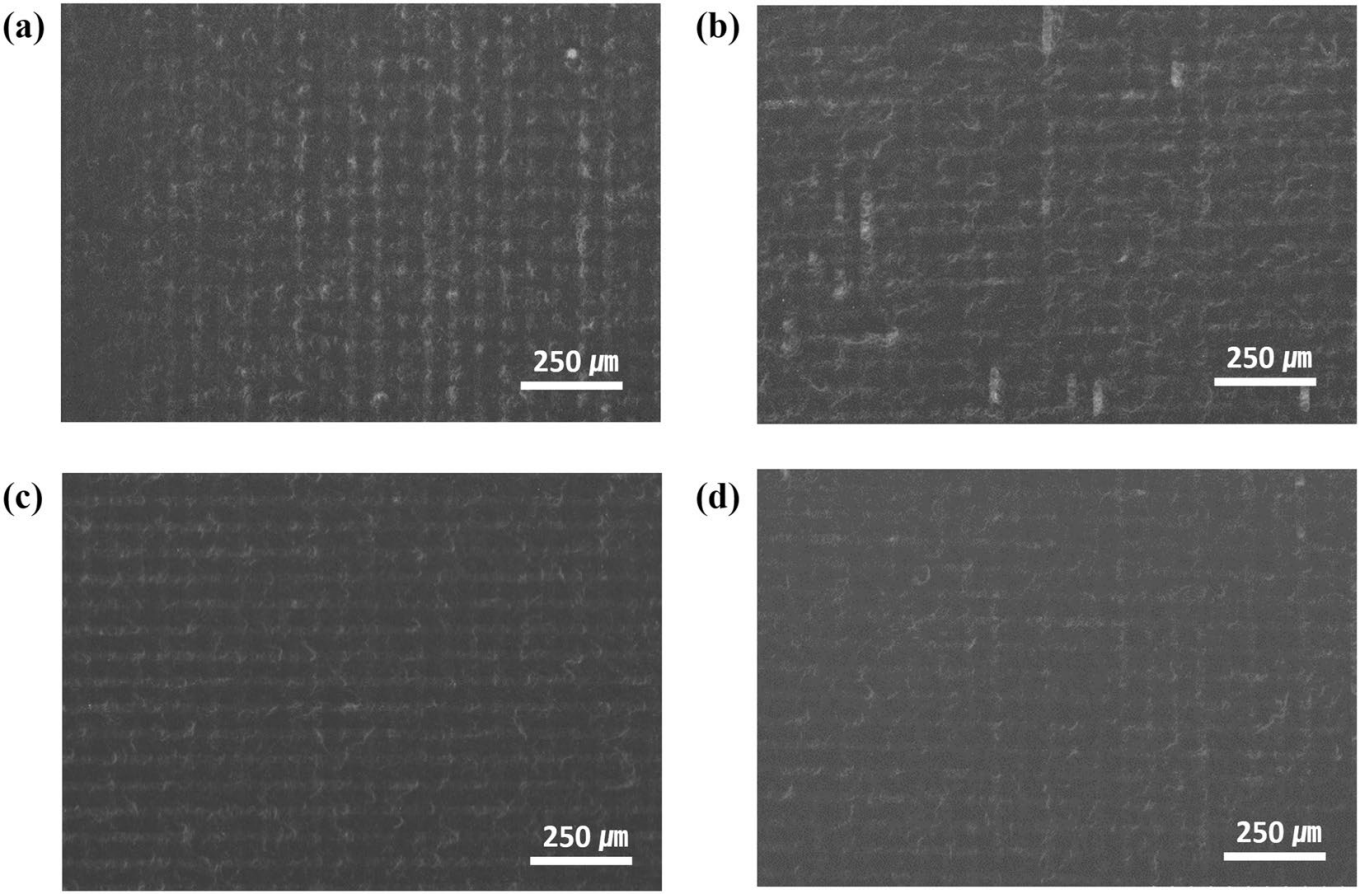
SEM images of hybrid Ag NW electrodes with grid width of 20 µm and grid pitch of 50 µm. Ag NWs spin-coated at (**a**) 2000, (**b**) 3000, (**c**) 4000, and (**d**) 5000 rpm on Ag NW grid patterns.

Second, to observe how the optoelectronic characteristics of a random network are affected by hybrid Ag NW electrode characteristics, Ag NW was spin-coated on an Ag NW grid with a grid pitch of 50 µm at varying spin-coating speeds. Figure 4 shows SEM images of hybrid Ag NW electrodes, and Table 1 summarizes the transmittances and sheet resistances of spin-coated and hybrid Ag NW electrodes. Because the density of the spin-coated Ag NW random network decreased with increasing spin-coating speed, both the transmittance and sheet resistance of the spin-coated Ag NW electrode increased with increasing spin-coating speed (Figure S3, SI). When Ag NW was spin-coated at 2000 rpm on Ag NW networks with a grid pitch of 50 µm, the transmittance of the hybrid Ag NW electrode slightly reduced (0.25%), while its sheet resistance reduced significantly (51.26%) when compared to the electrode containing only a randomly oriented network of Ag NWs. These results show that our Ag NW electrode fabrication method for obtaining hybrid networks consisting of both grid and random networks is novel, and can remarkably reduce sheet resistance because of the increased number of percolation paths, while minimizing the loss of transmittance.

**Table 1:** Transmittances and sheet resistances of Ag NW electrodes.

Sample		Transmittance (%)	Sheet resistance (Ω/☐)
Ag NW random network (spin-coated)	2000 rpm	97.2	99.0
	3000 rpm	98.2	117.3
	4000 rpm	98.3	160.5
	5000 rpm	98.8	201.3
Ag NW grid network + Ag NW random network (spin coated)	20 × 50 + 2000 rpm	97.0	48.3
	20 × 50 + 3000 rpm	97.9	68.8
	20 × 50 + 4000 rpm	97.9	112.8
	20 × 50 + 5000 rpm	98.3	130.5

**Figure 5:**
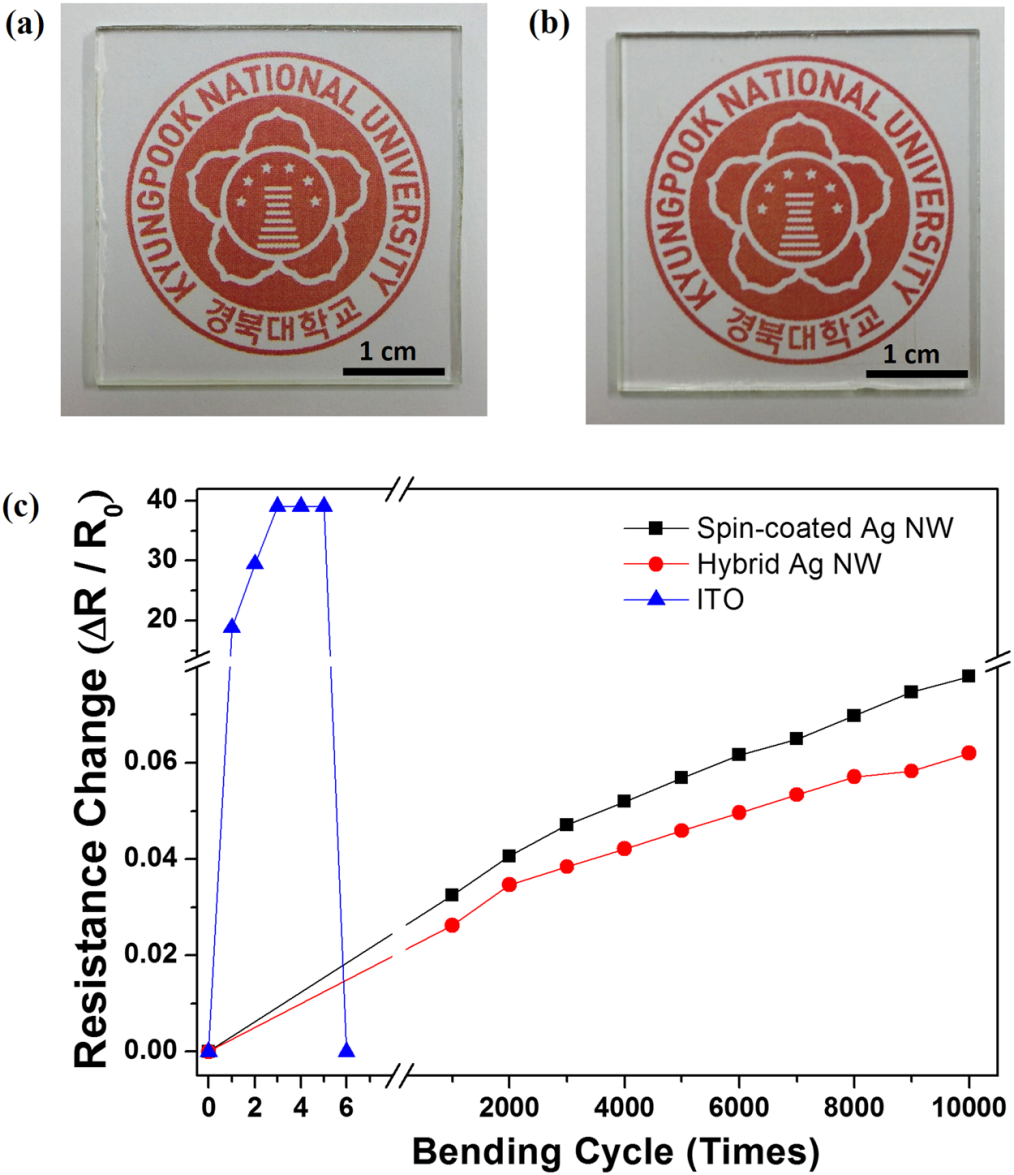
Photographs of (**a**) spin-coated Ag NW electrode and (**b**) hybrid Ag NW electrode on glass substrates. (**c**) Changes in the sheet resistances of spin-coated Ag NW network, hybrid Ag NW network, and ITO electrodes on PEN during bending tests.

Figure 5(a,b) show photographs of Ag NW electrodes obtained by spin-coating Ag NWs at 2000 rpm and hybrid Ag NWs with a grid pitch of 50 µm on glass substrates, respectively, placed on the logo of our institution. Compared to conventional ITO, the important advantages of Ag NWs are mechanical flexibility, high electric conductivity, and optical transmittance of the derived network. The mechanical flexibility and durability of the hybrid Ag NW electrode on polyethylene-naphthalate (PEN) were investigated using bending-fatigue equipment, by bending over a 5 mm radius of curvature at a speed of 1 cycle/s; the sheet resistances of the electrodes were compared to their initial values. For comparison, ITO and spin-coated Ag NW electrodes on PEN were subjected to bending tests. As shown in Fig. 5(c), the sheet resistance of the ITO electrode increased rapidly after several cycles, while changes in the sheet resistances of hybrid and spin-coated Ag NW electrodes were within 6% and 8%, respectively, of their initial values after 10,000 bending cycles. The change in sheet resistance of the hybrid Ag NW electrode after the bending test was considerably smaller than that of the ITO electrode.

**Figure 6:**
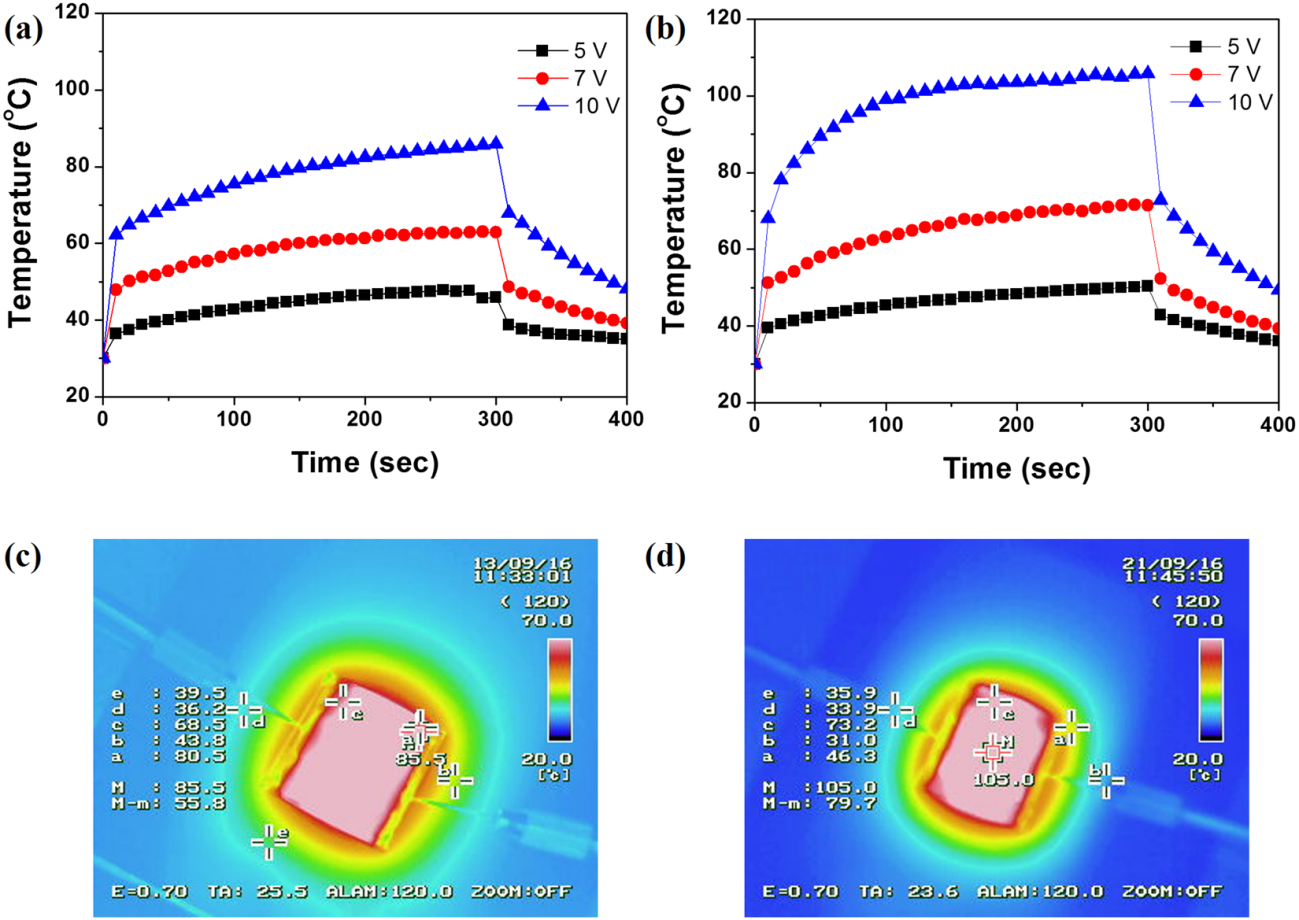
Temperature *vs*. time curves for (**a**) spin-coated Ag NW heater and (**b**) hybrid Ag NW heater on PEN substrates at applied voltages of 5, 7, and 10 V. Infrared (IR) images of (**c**) spin-coated Ag NW heater and (**d**) hybrid Ag NW heater at an applied voltage of 10 V.

To further evaluate the performances of hybrid Ag NW electrodes, we fabricated flexible transparent film heaters (TFHs) on PEN. Recently, TFHs have attracted growing interest for a wide range of applications: vehicular window defrosting, heating source for sensors, reaction cells, and heat retaining windows^21–24^. In particular, flexible TFHs are indispensable when anti-fogging and anti-icing characteristics are required to operate next-generation optoelectronic devices of irregular shapes on non-rigid and non-planar substrates. Changes in temperature–time profiles with various input voltages applied for 300 s to spin-coated and hybrid Ag NW TFHs are shown in Fig. 6(a–c), respectively. The temperature profiles show that the temperature of the hybrid Ag NW TFHs reached 54.6 and 77.2 °C when the input voltage was set at 5 and 7 V, respectively. Upon increasing the input voltage to 10 V, the temperature of the hybrid Ag NW TFH exceeded 110 °C, demonstrating good operation at low input voltages. Compared to the spin-coated Ag NW TFH operated at the same input voltage, the hybrid Ag NW TFH showed a higher maximum temperature. These trends are in accordance with the sheet resistances of the spin-coated and hybrid Ag NW electrodes, because thermal energy production is related to Joule’s law expressed as:

1 $$Q=\frac{{V}^{2}}{R}\times t$$

where *Q* is the heat produced, *V* is the applied potential, *R* is the resistance of the electrode, and *t* is the working time^25^. Moreover, the response time—defined as the time required to reach 90% of the maximum steady-state temperature—of the hybrid Ag NW TFH was ~100 s, suggesting fast response of the hybrid Ag NW TFH^26^. Moreover, infrared (IR) images (Fig. 6(c,d)) revealed a uniform temperature distribution in the hybrid Ag NW TFH, although a lower temperature was observed at the edges of the TFH due to the additional heat loss.

## Conclusions

We developed a method for fabricating hybrid Ag NW electrodes composed of randomly oriented and grid-patterned Ag NW networks. We first demonstrate a technique for fabricating the Ag NW grid pattern by repeating the MIMIC process twice, positioning the directions of the micro-channels perpendicular to each other. The effects of micro-channel heights and widths, and concentrations of the Ag NW suspensions on the morphologies of the Ag NW line patterns were investigated for Ag NW line patterns fabricated using the MIMIC process. Ag NW line patterns with various line widths could be fabricated with a 0.05 wt% Ag NW suspension. By spin-coating Ag NWs on a grid containing Ag NW networks, a hybrid Ag NW electrode was fabricated. The transmittance of the hybrid Ag NW electrode decreased slightly, while its sheet resistance decreased significantly relative to the electrode containing only a randomly oriented network of Ag NWs. For potential application of hybrid Ag NW electrodes in electronic devices, we also demonstrated flexible TFHs with fast response times and uniform temperature distributions.

This work demonstrates that hybrid Ag NW electrodes composed of randomly oriented and grid-patterned Ag NW networks are excellent candidates for low-cost, ITO-free TCEs in many applications, including optoelectronic devices and future flexible electronics. Although we have only demonstrated the use of a MIMIC process to fabricate Ag NW grid patterns in this study, various strategies for the preparation of Ag NW grids may be further explored and applied to fabricate hybrid Ag NW electrodes, which will lead to improved utility in the mass production of Ag NW TCEs for next-generation optoelectronic devices.

## Methods

### PDMS mold fabrication

SU-8 was spun onto a sacrificial layer and the substrate was subjected to pre-exposure baking on a hot plate. The SU-8 was exposed to UV light using a mask alignment system. Following this, post-exposure baking was conducted to complete the cross-linking process. The patterned structures were developed using an SU-8 developer to remove unexposed SU-8, and subsequently rinsed with isopropyl alcohol to remove any traces of the developer. Finally, PDMS molds with micro-channels were fabricated by casting a PDMS precursor (Sylgard184, Dow Corning) against a completed SU-8 relief structure.

### Hybrid Ag NW electrode fabrication

The PDMS mold was attached to the substrate. To obtain micro-channels, the patterned surface of the PDMS mold was brought into contact with the substrate surface. When the Ag NW suspension was introduced at the PDMS mold tip, the suspension flowed spontaneously into the micro-channels due to capillary action. After annealing at 65 °C for 1 h, Ag NW line patterns were obtained by releasing the PDMS mold from the substrate. To fabricate the Ag NW grid pattern, the micro-channel of the PDMS mold was placed on the substrate in a direction perpendicular to the Ag NW line patterns fabricated by the first MIMIC process. A second MIMIC process was performed to form Ag NW line patterns aligned in a direction perpendicular to the initial Ag NW line patterns. Finally, hybrid Ag NW electrodes comprising both random and grid networks were obtained by spin-coating a 0.3 wt% Ag NW suspension onto the Ag NW grid.

### Characterization

The morphologies of Ag NW were characterized by SEM (SU8220, Hitachi). Optical transmittance spectra were measured using a UV-VIS spectrometer (UV-160A, Shimadzu). Sheet resistances were measured using a four-point probe technique. Heaters worked at a constant voltage supplied by a power source (model 2400, Keithley). The temperatures of the heaters were measured with an IR camera (G100EX, Avio).

## Electronic supplementary material

Supplementary information
