# Supplementary material for: Hybrid Ag nanowire transparent conductive electrodes with randomly oriented and grid-patterned Ag nanowire networks
Source: Sci Rep. 2017 Sep 14;7:11614. doi: 10.1038/s41598-017-11964-w (PMC5599587; doi:10.1038/s41598-017-11964-w)
Supplement: Supplementary file 1 — Supplementary information [file 41598_2017_11964_MOESM1_ESM.doc]

**Supplementary Information**

**Hybrid Ag nanowire transparent conductive electrodes with randomly oriented and grid-patterned Ag nanowire networks**

**Bonhee Ha and Sungjin Jo^*^**

School of Architectural, Civil, Environmental, and Energy Engineering, Kyungpook National University, Daegu 41566, Korea

*E-mail: sungjin@knu.ac.kr


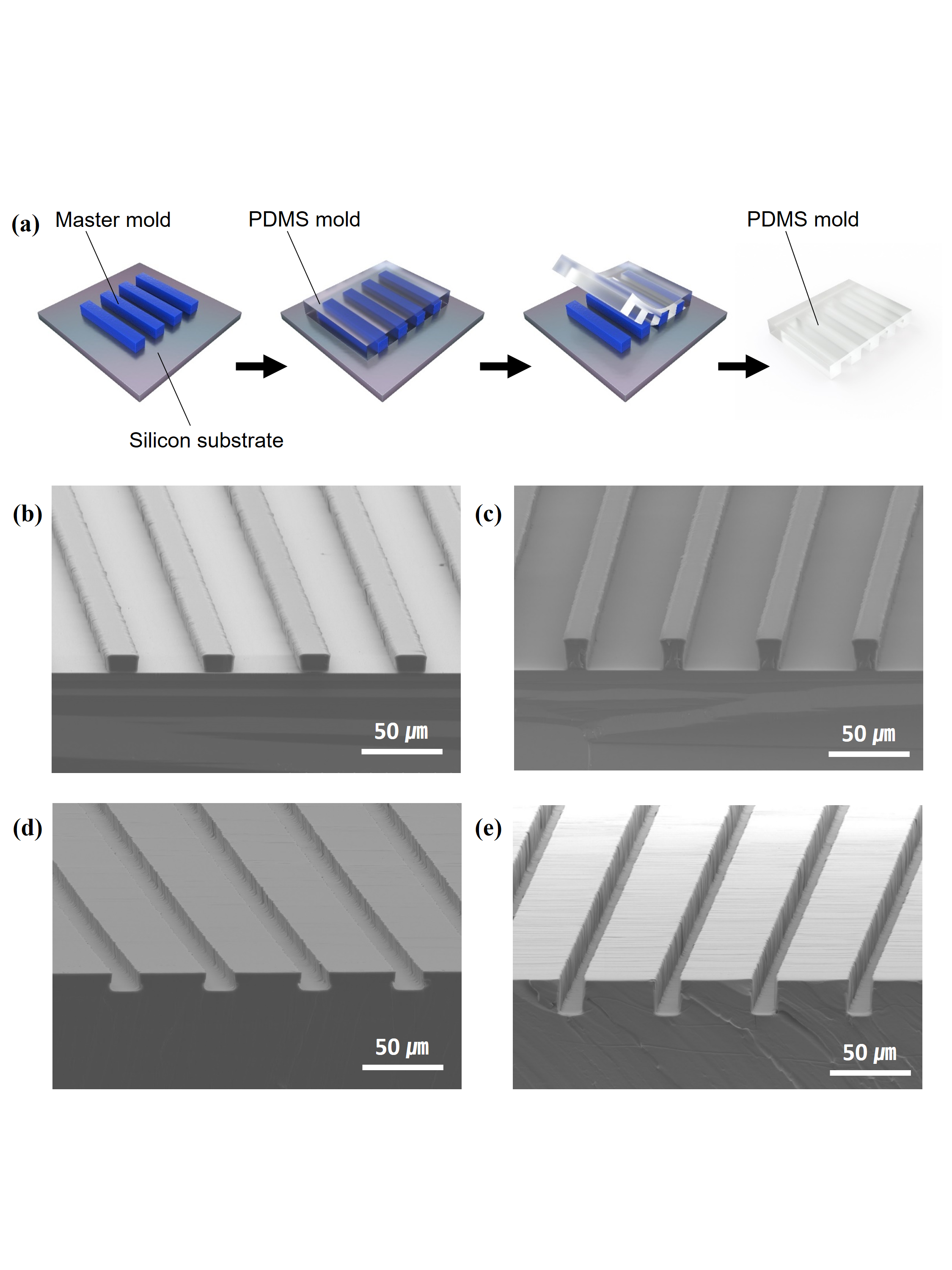


**Figure S1.** Schematic illustration of the steps involved in fabricating the PDMS mold used for the MIMIC process. SEM images of SU-8 line patterns on Si wafer fabricated by (b) SU-8 2007 and (c) SU-8 2025; (d) and (e) SEM images of PDMS molds replicated from (b) and (c), respectively.


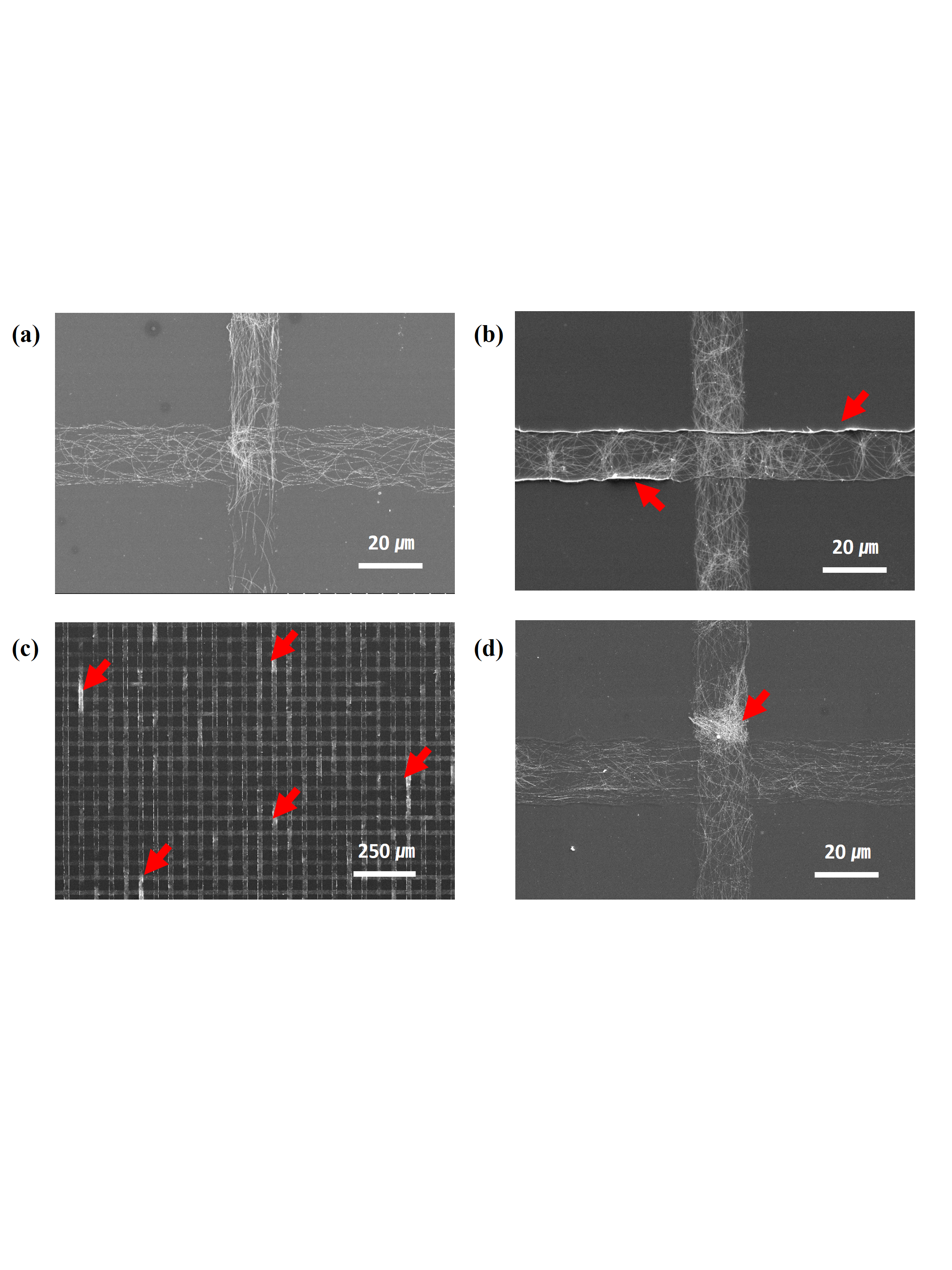


**Figure S2.** SEM images of Ag NW grid patterns fabricated with micro-channel heights of (a) 7 µm and (b) 25 µm. (c) SEM image of the Ag NW grid patterns fabricated using 3 wt% Ag NW suspension. (d) Magnified SEM image of (c). Arrow indicates an uneven grid pattern due to aggregation of Ag NWs.


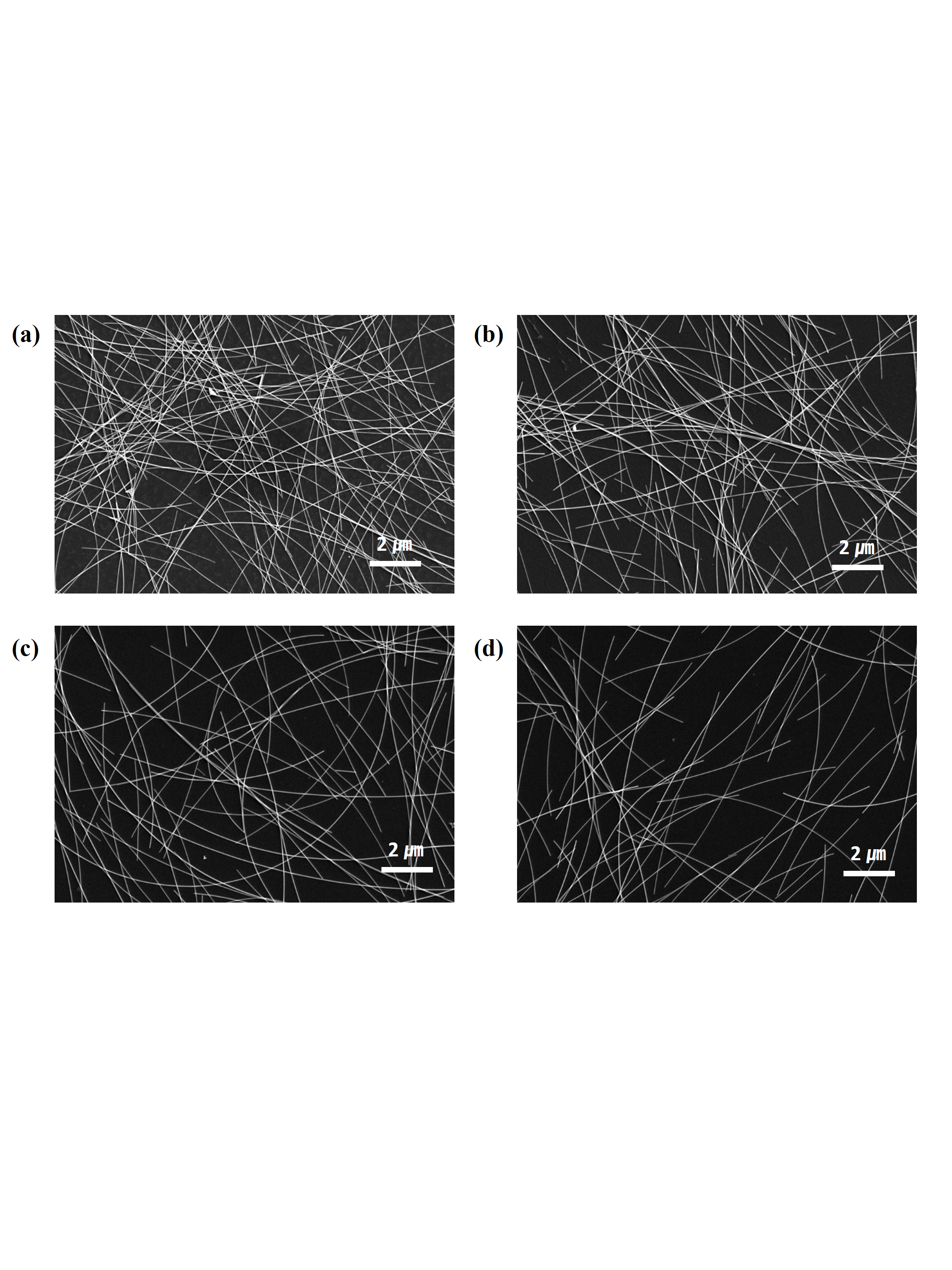


**Figure S3.** SEM images of Ag NWs spin-coated at (a) 2000 rpm, (b) 3000 rpm, (c) 4000 rpm, and (d) 5000 rpm.


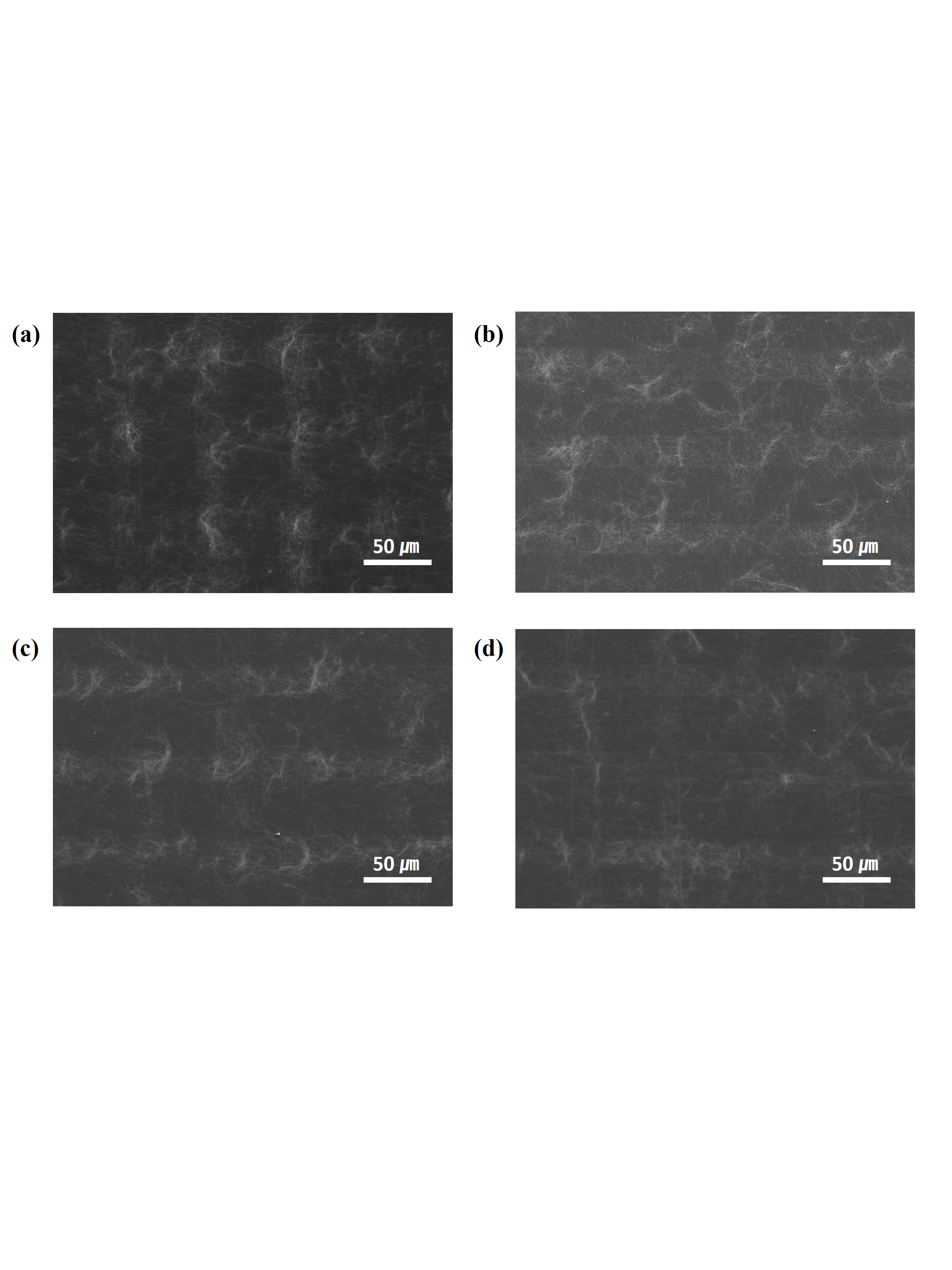


**Figure S4.** SEM images of hybrid Ag NW electrodes with grid width of 20 µm and grid pitch of 50 µm. Ag NWs spin-coated at (a) 2000 rpm, (b) 3000 rpm, (c) 4000 rpm, and (d) 5000 rpm on Ag NW grid patterns.

**Table S1.** Transmittances and sheet resistances of Ag NW electrodes.

| Sample | | Transmittance  (%) | Sheet resistance  (Ω/□) |
| --- | --- | --- | --- |
| Ag NW random network  (spin-coated at 4000 rpm) | | 98.6 | 198.0 |
| Ag NW grid network | 20 × 50 | 99.44 | - |
|  | 20 × 200 | 99.75 | - |
|  | 20 × 400 | 99.92 | - |
| Ag NW grid network  +  Ag NW random network  (spin-coated at 4000 rpm) | 20 × 50 | 98.3 | 127.3 |
|  | 20 × 200 | 98.3 | 157.5 |
|  | 20 × 400 | 98.5 | 181.7 |

**Table S2.** Haze factors of hybrid Ag NW electrodes.

| Sample | | Haze  (%) |
| --- | --- | --- |
| Ag NW grid network  +  Ag NW random network  (spin-coated) | 20 × 50 + 2000 rpm | 0.49 |
|  | 20 × 50 + 3000 rpm | 0.46 |
|  | 20 × 50 + 4000 rpm | 0.42 |
|  | 20 × 50 + 5000 rpm | 0.37 |
